# Partial Nephrectomy in Solitary Kidneys: Intraoperative Techniques and Their Impact on Chronic Kidney Disease Progression

**DOI:** 10.3390/cancers18101644

**Published:** 2026-05-19

**Authors:** Benjamin N. Schmeusser, Courtney Yong, Daniel Sidhom, Edouard H. Nicaise, Reza Lahiji, James E. Slaven, Dattatraya H. Patil, Kenneth Ogan, Chandru Sundaram, Viraj A. Master, Ronald S. Boris

**Affiliations:** 1 Department of Urology, School of Medicine, Indiana University, 535 Barnhill Drive|RT 150, Indianapolis, IN 46202, USA; cyong@iu.edu (C.Y.); rboris@iuhealth.org (R.S.B.); 2 Department of Urology, Lenox Hill Hospital, New York, NY 10075, USA; edouard.h.nicaise@gmail.com; 3 Department of Urology, Emory University, Atlanta, GA 30322, USA; reza.lahiji@emory.edu (R.L.);; 4 Department of Biostatistics and Health Data Science, Indiana University, Indianapolis, IN 46202, USA

**Keywords:** renal cell carcinoma, partial nephrectomy, solitary kidney, renal function

## Abstract

Objective: Solitary kidney tumors are a challenging scenario necessitating both oncologic efficacy and renal function preservation. Partial nephrectomy (PN), when feasible, remains the gold standard for management. We examine intraoperative techniques and their association with renal function decline in patients with solitary kidneys undergoing PN. Methods: In two high volume academic referral centers, we analyzed patients that underwent PN in a solitary kidney from 2000 to 2023. Patient characteristics, tumor details, and operative details were obtained. Chronic kidney disease (CKD) upstaging from pre- to postoperative was the primary outcome, with multivariable analysis examining the association between intraoperative factors and CKD progression. Results: In total, 104 patients were included, of which 38 (36.5%) experienced CKD upstaging. Mean eGFR decline was 15.4% at median follow-up of 16 months. Cold ischemia was associated with higher odds of CKD upstaging compared to warm ischemia (OR 3.64; 95% CI 1.06–12.52) and no ischemia (4.55; 95% CI 1.09). Notably, cold ischemia cases tended to involve significantly larger, more complex tumors in patients with lower baseline renal function. Ischemia time, parenchyma resection, renal volume change, operative time, and renorrhaphy type were not predictors of CKD upstaging. Conclusions: PN in solitary kidneys remains standard with evidence of excellent renal preservation in this cohort. Worse outcomes were observed with cold ischemia, although this more likely represents underlying tumor complexity with other uncontrollable factors; however, this should be explored further. These findings suggest that renal functional outcomes are likely reasonable in patients with solitary kidneys undergoing PN when appropriate patient selection and sound surgical technique are utilized.

## Simple Summary

A kidney tumor in a patient with only one functioning kidney is a unique surgical challenge. The tumor must be removed while preserving healthy kidney tissue to avoid dialysis. Partial nephrectomy is preferred because it removes only the tumor and as little surrounding tissue as possible. However, it is unclear if specific surgical techniques influence the long-term kidney function of these patients. This study considers over 100 patients at two major medical centers that underwent partial nephrectomy on a solitary kidney. The techniques used in the operating room, such as blood flow interruption method, amount of tissue removed, and closure technique, and the impact on kidney function were considered. These findings suggest that partial nephrectomy in solitary kidneys can achieve favorable renal functional outcomes with appropriate patient selection and sound surgical technique, but future study of certain techniques, such as the use of ice in the surgery, is warranted.

## 1. Introduction

A renal mass in a solitary kidney (RMSK) is a challenging urologic scenario. Goals of management include optimization of renal function while maintaining oncologic control and therefore represents an absolute indication for a nephron sparing approach [1]. While active surveillance or percutaneous interventions such as thermal ablation are viable options, partial nephrectomy (PN) is the preferred treatment for RMSK [2].

The feasibility of PN for the treatment of RMSK is well established [2,3,4,5,6,7,8]. These studies highlight not only satisfactory oncologic control but also reasonable renal function preservation, with an expected postoperative decline in renal function estimated of 20% [9]. Despite this, there is continued emphasis on refining surgical technique and implementing operative maneuvers to minimize renal function loss as much as possible. Evidence suggests that maintenance of healthy renal parenchyma and minimization of ischemia damage are core operative principles for preserving renal function following PN, in addition to non-modifiable factors such as age, comorbidities, and preoperative eGFR [9,10].

In this study, we examine the association between intraoperative techniques and surgical factors on renal function outcomes following PN for RMSK in a multi-institutional cohort. We first examine the overall functional outcomes following PN in these patients. We then consider whether intraoperative techniques such as ischemia time and type, renal parenchyma loss, renorrhaphy type, and other aspects contribute to a greater chance of chronic kidney disease (CKD) upstaging.

## 2. Methods

### 2.1. Study Design and Patient Selection

Following IRB approval, databases at Indiana University (IRB 20376) and Emory University (IRB 00055316) were reviewed for patients that underwent PN for RMSK from 2000 to 2023. Both institutions performed more than 1300 PN over the past 10–15 years, with included surgeons generally performing more than 30 annually. Demographics and comorbidities were collected. Estimated glomerular filtration rate (eGFR) was calculated using the recommended 2021 chronic kidney disease epidemiology collaboration (CKD-EPI) creatinine-based equation, which excludes race as a cofactor, and grouped into CKD stages [11,12]. Patients with missing data were excluded. Further information included surgical details, such as tumor size (cm); tumor complexity as determined by their RENAL nephrometry score (Radius, Exophytic/endophytic, Nearness to collecting system, Anterior/posterior, and Location to polar line) [13]; surgical approach; operative time (min), ischemia time and type (warm, cold, none); and renorrhaphy 1 vs. 2 layers. Pathologic data was collected, including tumor stage, histology, and parenchyma pathology (healthy vs. unhealthy). Follow-up data included postoperative eGFR and CKD stage, volume of parenchyma resected, weeks to postoperative imaging, and need for postoperative dialysis at any point. eGFR at last follow-up was used as the postoperative eGFR. Kidney volume was calculated using computed tomography for pre- to post-solitary kidney PN volume change [14]. Pathology reports were reviewed to assess resected parenchyma volume based on gross measurements of normal parenchyma within specimens.

### 2.2. Statistical Analysis

The primary outcome of the study was to determine intraoperative factors that may contribute to patients experiencing an upstaging of their CKD stage following PN for RMSK. The cohort was dichotomized based on their CKD stage increasing (i.e., stage 2 to 3a) or if they remained stable post PN. Groups were compared. Multivariable analysis was performed to assess for intraoperative factors and their ability to predict CKD upstaging. Analysis was additionally conducted to determine cohort differences based on ischemia type used intraoperatively.

Quantitative data were reported as means/standard deviations or medians/ interquartile ranges. Qualitative data were described as numbers and percentages. Cohorts were compared using Chi-Square tests (Fisher’s Exact where appropriate) and Wilcoxon tests. Bivariable and multivariable logistic regression models were conducted with upstaging in CKD stage as the outcome of interest. Multivariable/adjusted models controlled for age, body mass index, hypertension, diabetes, RENAL score, and presence of unhealthy parenchyma on pathology. Values were presented as odds ratios with 95% confidence intervals and *p*-values. All statistical tests were two-sided, with type I error set at 0.05. Analyses were performed using SAS v9.4 (SAS Institute, Cary, NC, USA), with all analytic assumptions being verified.

## 3. Results

Our patient cohort is presented in Table 1. In total, 104 patients were included, 38 (36.5%) of which experienced an increase in their CKD stage. Mean preoperative eGFR was 55.9 (95% CI 51.8–60.1, SD 21.6) and mean postoperative eGFR at a median follow-up time of 16 months was 51.9 (95% CI 46.8–56.9, SD 26.0), which was notable for a mean decline of about 15.4%. A majority of the patients were male (61.5%) and white (80.8%). Average eGFR preoperative was 51 (CKD stage 3a). The average mass was moderately complex with a RENAL score of 8. Most cases were open (66.4%); however, there was an overall trend towards increased robotics, as only 58% of cases were open from 2020 to 2023. Median ischemia time was 17 min with no ischemia in 23.3% of cases. Cold ischemia was used in 42.7% of patients and warm ischemia in 34.0%. Most surgeons used 2 layers for renorrhaphy (66.3%). Pathologically, clear cell renal cell carcinoma was the predominant histology (71.2%), and most patients had a T1 (59.6%) or T2 (26.9%) mass. Average parenchyma volume was 100.7 mL, and parenchyma being considered unhealthy was found in 46.2% of patients. Postoperatively, 12 (11.5%) of patients required dialysis at some point, with only three of these patients having persistent end-stage renal disease after long-term follow-up. There was a recurrence rate of 26.9% at 5 years.

Table 1 subdivides our cohort based on CKD upstaging vs. stable-to-improved CKD staging. An increased incidence of CKD upstaging was observed in patients that underwent an open approach (79% vs. 59.1%, *p* = 0.039). Cold ischemia had higher rates of CKD upstaging (62.2% vs. 31.8%, *p* = 0.011). An increase in CKD stage was more likely in T3 tumors (26.3% vs. 6.1%, *p* = 0.014) and when more parenchyma was resected (129 vs. 79.5, *p* = 0.014).

**Figure 1:**
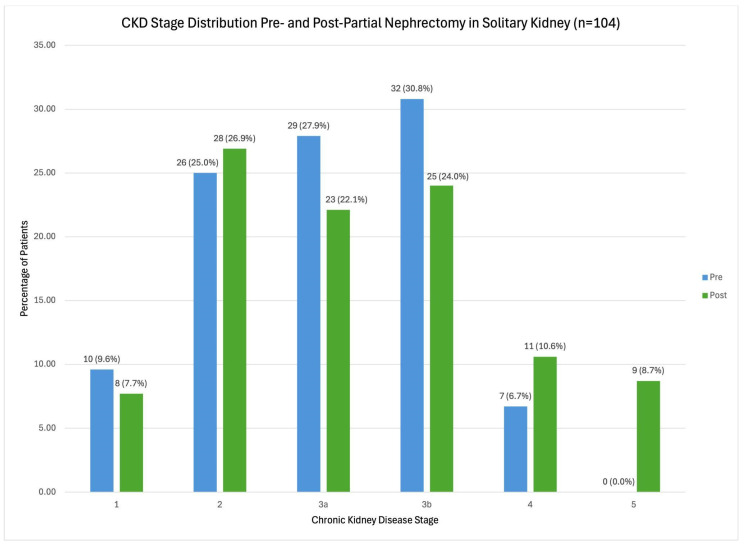
Distribution of chronic kidney disease stages pre- and post-partial nephrectomy in solitary kidneys (*n* = 104). There was an increase in patients with CKD stage 2, 4, and 5 following partial nephrectomies. Pre-partial nephrectomy is represented by blue bars, and post-partial nephrectomy represented by green bars. Total number of patients and corresponding percent at each chronic kidney disease stage is labeled (*n* [%]).

The distribution of pre- and post-PN CKD stages is presented in Figure 1. In general, most patients were in stages CKD2, 3a, and 3b preoperatively. From pre- to post-PN, an increased percentage of patients was found in CKD stages 2, 4, and 5, but a decrease percentage in CKD stages 1, 3a, and 3b.

Results from our univariable and multivariable models looking at intraoperative factors are presented in Table 2. Our models controlled for patient factors such as age, body mass index, hypertension, diabetes, nephrometry score, and presence of unhealthy parenchyma on pathology. Ischemia time, renal parenchyma resected, change in renal volume, operative time, and renorrhaphy layers (1 vs. 2) did not appear to significantly influence postoperative CKD staging. The risk of CKD progression was higher in patients that underwent cold ischemia as compared to warm ischemia (OR 3.64 [1.06–12.52], *p* = 0.041) and compared to no ischemia (OR 4.55 [1.09–18.98], *p* = 0.038).

Given the apparent association with ischemia type, a comparison of patients that underwent no ischemia (*n* = 24), cold ischemia (*n* = 44), or warm ischemia (*n* = 35) is presented in Table 3. Overall, these groups were similar at baseline. The cold ischemia group did have lower preoperative GFR by about 10 points although statistically insignificant. Patients that underwent cold ischemia had tumors that were larger (4.3 cm vs. 2.9 cm [none] vs. 3.2 cm [warm], *p* ≤ 0.001), more likely to be T2 or T3 stage (*p* < 0.001), and more complex (RENAL score 8.5 vs. 6.5 [none] vs. 7 [warm], *p* = 0.002). All cold ischemia cases were open as compared to 54% of no ischemia and 31% of warm ischemia cases (*p* < 0.001). Operative times and renorrhaphy layers were similar. Less parenchyma was resected in the cold ischemia group (16.7 vs. 26.1 [none] vs. 86.3 [warm], *p* < 0.001).

## 4. Discussion

In this study, we considered the impact of intraoperative techniques on the kidney function following PN for RMSK in a multi-institutional cohort. Overall ischemia time, parenchyma resected, operative time and renorrhaphy layers did not appear to significantly influence renal function outcomes. This cohort experienced favorable renal preservation with an average decline of only about 15%. There was an apparent association between cold ischemia type and worsening renal function; however, these cases tended to involve larger, more complex renal masses and had lower preoperative eGFR values that likely confound the results. Prior larger series primarily describe overall renal functional outcomes after partial nephrectomy in solitary kidneys. Alternatively, our study focuses on the independent impact of modifiable intraoperative factors using CKD upstaging, a more clinically meaningful and actionable endpoint given distinct management implications [15,16,17,18]. These findings suggest that, within modern practice, overall surgical judgment and baseline disease factors may play a greater role than specific intraoperative maneuvers and that good clinical judgment with sound surgical technique will likely result in overall favorable functional outcomes for these high-risk patients.

PN represents the standard for resectable renal masses, including more complex larger masses [19], and is critically crucial for RMSK [1]. While successfully removing the mass and ensuring an optimal oncologic outcome is ideal, urologists should weigh the risk of declining renal function, which has associated negative impact on health, quality of life, and future treatment options [20]. In patients with bilateral kidneys, the literature suggests around a 10% loss in overall renal function following PN, with evidence of nearly 20% decline in the function of the kidneys operated on specifically [9,21,22]. This decline in renal function is typically minimized due to continued function and postoperative compensatory hypertrophy of the contralateral kidney [23]. Renal function preservation is closely linked to renal quantity and quality [24]. Therefore, a greater decline is expected with a larger resection of non-tumor parenchyma; an incomplete recovery of nephrons following surgical factors such as ischemia; and other patient specific factors such as increased age, additional medical comorbidities, and lower baseline preoperative eGFR [9,10,25]. This was consistent with our results showing patients with larger, more complex masses and greater amounts of parenchyma resected experienced greater declines as compared to less complex, smaller exophytic masses.

The impact of PN on renal function has been explored in patients with solitary kidneys. In over 800 patients with that underwent PN for RMSK, an average renal function loss of about 20% was found, although this varied depending on various intraoperative features such as ischemia time and type and parenchymal preservation [2,3]. Our cohort had a mean decline of 15%, a favorable finding that may be explained by being a more contemporary cohort and most surgeries being performed by surgeons with a high volume in PN practice. Although not significant in our cohort, a greater decrease in parenchymal volume and longer ischemia times have been previously associated with greater declines in renal function [3,4]. The lack of significance with ischemia time observed in our study is likely attributable to the low median ischemia time (17 min) in our group, consistent with the literature suggesting ischemia time likely has minimal long-term impact when kept within reason (i.e., 30 min) [9,26,27]. Increased parenchyma resected, although not significant after adjusting for confounders, did have a significantly greater amount of patients in the CKD upstaging cohort. With regard to renorrhaphy, we did not find any significant association between 1- and 2-layer closures, similar to other published studies [28,29], but other studies have found renal function preservation with the single-layer closure [30]. Although conflicting, this does appear to be the first study to consider this for RMSKs.

Interestingly, our study found a higher risk of CKD upstaging with cold ischemia. This varies from the existing literature. Lane et al. analyzed 660 patients with solitary functioning kidneys from several institutions from 1980 to 2009. Their cohort was fairly evenly split between cold/warm ischemia times, and they found 3-month GFR similar despite longer cold ischemia times (45 vs. 22 min) [8]. Funahashi et al. utilized renal function scans post PN in 59 patients with warm ischemia and 64 patients with cold ischemia. Preservation of renal function in the cold ischemia group was observed, even up to an hour of ischemia time, whereas warm ischemia greater than 25 min was detrimental [31]. Alternatively, Gurram et al. analyzed 700+ patients that underwent PN and found patients that received cold ischemia experienced the greatest decline in renal function at 12 months; however, this did not persist after controlling for other factors [32]. While our results showed greater CKD upstaging with cold ischemia, there are significant limitations, including that the study was not aiming to answer this specific question. As shown, these patients had much larger and more complex renal masses with lower preoperative eGFR (although statistically not significant), and often underwent longer ischemia times. RENAL scores in our analysis should help account for mass size and complexity; however, there are likely further confounders unable to be accounted for this, making it difficult to compare these cohorts fairly. Alternatively, it may be that among patients with more vulnerable solitary kidneys, ischemia type and time carry greater significance as compared to patients with two kidneys.

However, a smaller subset of our patients (*n* = 24) with no ischemia is worth considering. Through techniques such as tumor enucleation, urologists may attempt a PN “off-clamp” to minimize the potential negative impacts of ischemia [33]. Our results indicate safety and feasibility with renal function loss comparable to patients with warm ischemia; however, these patients had smaller, less complex masses, and it is again worth noting that our ischemia time on average was <20 min. Calaway et al. examined 12 patients undergoing PN for RMSK, 6 of which underwent no ischemia and 6 of which had an average of only 5.5 min of ischemia [34]. Their findings were encouraging, with 0% positive margins and a mean loss of renal function of only 7% [34]. Furthermore, in the aforementioned study by Attawettayanon et al., 5% (*n* = 45) of their cohort was managed with zero ischemia. While these patients had significantly smaller and less complex masses, they reported a preserved renal function of 99% [3]. Alternatively, a more recent study by Gurram et al. examined over 700 PNs and found no significant difference in long-term renal function for patients that experienced no ischemia (*n* = 455), cold ischemia (*n* = 63), warm ischemia < 30 min (*n* = 164), and warm ischemia > 30 min (*n* = 60) [32]. Furthermore, Cignoli et al. considered this idea in 863 patients undergoing PN, 277 of which underwent no ischemia [35]. Their findings were less encouraging for the implementation of such techniques, as they found no ischemia or short ischemia times to be associated with a higher risk of bleeding complications without any significant improvement in long-term renal function [35]. Although specific indications for zero ischemia and techniques, such as tumor enucleation for PN, are not yet clearly defined, evolving evidence suggests that in experienced hands it is likely safe and may be useful in scenarios like RMSK, where preserving renal function is imperative. Surgeons considering PN in a solitary kidney should have familiarity with techniques such as tumor enucleation to maximize efficacy in this challenging space.

While these results are informative on renal function outcomes in patients undergoing PN for RMSK, there are certainly limitations and important considerations. As a retrospective study, it is subject to biases such as selection bias and other issues such as inaccurate or missing data. Examining CKD upstaging as the primary endpoint may be more clinically relevant given the implications of different CKD stages; however, it should be recognized that patients may experience varying declines in function without being upstaged. Calculation of parenchyma resection by pathologic assessments are limited by pathologist variation and specimen presentation (i.e., no fat vs. fat present), and volume changes on imaging are limited by individual measuring and imaging processing. Inclusion of patients with complex and often large renal masses may confound results, so future considerations in larger cohorts may include focusing on different T-stages. Oncologic outcomes were not a primary endpoint; however, the 5 year recurrence rate of 26.9% aligns with prior reports of 28% and 37% at 5 and 10 years, respectively [36]. Future directions may include further analysis of oncologic and survival outcomes in this high-risk population often impacted by familial syndromes and prior malignancy. Follow-up variance may limit data. The lack of controls limits interpretation of these results. This study is multi-institutional, which leads to greater variation in surgeon techniques and differences in data collection. There is a lack of generalizability given that both institutions are referral centers, with the included surgeons having significant experience in managing renal tumors. Finally, given the study spanned over 20 years, significant changes in operative technique and expertise may be unaccounted for.

## 5. Conclusions

We present the impact of several intraoperative factors CKD upstaging in patients undergoing PN in solitary kidneys in a large multi-institutional cohort. Overall, ischemia time, parenchyma resected, operative time, and renorrhaphy layers did not appear to significantly influence renal function outcomes. Cold ischemia did appear to negatively impact renal function; however, these results should be interpreted with caution and explored further, given that these renal masses were typically larger and more complex. These findings suggest good clinical judgment and sound surgical technique will likely result in overall favorable functional outcomes for these high-risk patients.

## Figures and Tables

**Table 1:** Demographics of patients that underwent partial nephrectomy in a solitary kidney (*n* = 104) further subdivided by patients that experienced CKD upstaging (*n* = 38) and patients that had stable-to-improved CKD staging (*n* = 66).

	Overall	CKD Upstaging	CKD Stable/ Improved	*p*-Value
**Age**	65 (27–85)	66.5 (35–82)	65 (27–85)	0.22
**Sex (*n* [%])**				
Female	40 (38.5)	17 (44.7)	23 (34.9)	0.32
Male	64 (61.5)	21 (55.3)	43 (65.2)	
**Race**				
Non-White	20 (19.2)	9 (23.7)	11 (16.7)	0.38
White	84 (80.8)	29 (76.3)	55 (83.3)	
**BMI**	30.1 (18.7–55.7)	30.1 (22.4–52.2)	29.9 (18.7–55.7)	0.42
**HTN**	75 (72.1)	28 (73.7)	47 (71.2)	0.79
**DM**	22 (21.2)	10 (26.3)	12 (18.2)	0.33
eGFR Preop *	51.5 (16.8–112.6)	45.7 (16.8–112.6)	54.0 (20.6–107.6)	0.14
**Tumor Size (cm)**	3.5 (1.4–12.5)	3.7 (1.7–12.5)	3.3 (1.4–10.3)	0.11
**RENAL Score**	8 (4–11)	7 (4–11)	7 (4–11)	0.18
**Surgical Approach**				
Robotic	35 (33.7)	8 (21.1)	27 (40.9)	**0.04**
Open	69 (66.4)	30 (79.0)	39 (59.1)	
Operative Time *	210 (66–419)	214.5 (66–362)	206 (67–419)	0.56
**Ischemia**				
Overall *	17 (0–86)	16 (0–86)	17 (0–64)	0.77
No Ischemia	24 (23.3)	6 (16.2)	18 (27.3)	**0.01**
Cold Ischemia	44 (42.7)	23 (62.2)	21 (31.8)	
Warm Ischemia	35 (34.0)	8 (21.6)	27 (40.9)	
**Renorrhaphy**				
**1 Layer**	29 (30.5)	9 (26.5)	20 (32.8)	0.80
**2 Layers**	63 (66.3)	24 (70.6)	39 (63.9)	
**Other/None**	3 (3.2)	1 (2.9)	2 (3.3)	
**T-Stage**				
T1	62 (59.6)	20 (52.6)	42 (63.6)	**0.01**
T2	28 (26.9)	8 (21.1)	20 (30.3)	
T3	14 (13.5)	10 (26.3)	4 (6.1)	
**Histology**				
ccRCC	74 (71.2)	27 (71.1)	47 (71.2)	0.85
pRCC	11 (10.6)	3 (7.9)	8 (12.1)	
chRCC	3 (2.9)	1 (2.6)	2 (3.0)	
Other	16 (15.4)	7 (18.4)	9 (13.6)	
**Parenchyma Pathology**				
Healthy	50 (53.8)	16 (47.1)	34 (57.6)	0.32
Unhealthy	43 (46.2)	18 (52.9)	25 (42.4)	
Weeks to Postop Scan *	7 (2–70)	7 (2–37)	7 (2–70)	0.55
**Resection Volume**				
Parenchyma Resected	100.7 (0–1730.7)	129.0 (0–1730.7)	79.5 (0–1474.7)	**0.01**
Parenchyma Change *	−57.6 (−625.0, 181.6)	−49.3 (−625.0, 181.6)	−57.7 (−277.3, 129.5)	0.85
**Postop Dialysis**				
No	92 (88.5)	27 (71.1)	65 (98.5)	**<0.001**
Yes	12 (11.5)	11 (29.0)	1 (1.5)	

Abbreviations: body mass index (BMI); hypertension (HTN); diabetes mellitus (DM); estimated glomerular filtration rate (eGFR); renal nephrometry score (RENAL); clear cell, papillary, and chromophobe renal cell carcinoma (ccRCC, pRCC, and chRCC). * indicates median (IQR). Values are frequencies (percentages) for categorical variables and medians (ranges) for continuous variables, with *p*-values from Chi-Square tests (Fisher’s Exact where appropriate) and Wilcoxon tests. **Bold** represents significance with *p*-value < 0.05.

**Table 2:** Influence of intraoperative factors on chronic kidney disease following partial nephrectomy on solitary kidneys.

Factor	Unadjusted	Adjusted
Ischemia time (min)	1.01 (0.99, 1.04); *p* = 0.27	1.01 (0.98, 1.04); *p* = 0.49
Renal parenchyma resected (100 units)	1.11 (0.99, 1.25); *p* = 0.06	1.10 (0.96, 1.26); *p* = 0.16
Change in renal volume	1.00 (1.00, 1.00); *p* = 0.61	1.00 (1.00, 1.01); *p* = 0.72
Procedure length (30 min)	1.03 (0.85, 1.25); *p* = 0.76	0.89 (0.69, 1.15); *p* = 0.37
Renorrhaphy layers 1 vs. 2	0.73 (0.29, 1.87); *p* = 0.51	0.65 (0.19, 2.22); *p* = 0.49
Ischemia type		
Cold (vs. none)	3.29 (1.10, 9.84); *p* = **0.03**	4.55 (1.09, 18.98); *p* = **0.04**
Warm (vs. none)	0.89 (0.26, 3.00); *p* = 0.84	1.25 (0.29, 5.39); *p* = 0.76
Cold (vs. warm)	3.70 (1.38, 9.91); *p* = **0.01**	3.64 (1.06, 12.52); *p* = **0.04**

Values are odds ratios (95% CIs) with *p*-values from (multiple) regression models, modeling CKD change > 0 (upstaging); *p*-value < 0.05 indicates statistical significance in **bold**. Multivariable/adjusted models include age, body mass index, hypertension, diabetes, renal score, and presence of unhealthy parenchyma on pathology.

**Table 3:** Demographics of patients by ischemia groups.

	None (*n* = 24)	Cold (*n* = 44)	Warm (*n* = 35)	*p*-Value
**Age**	68 (30–77)	65 (43–85)	63 (27–83)	0.08
**Sex (*n* [%])**				
Female	8 (33.3)	18 (40.9)	13 (37.1)	0.8
Male	16 (66.7)	26 (59.1)	22 (62.9)	
**Race**				
Non-White	5 (20.8)	11 (25.0)	3 (8.6)	0.16
White	19 (79.2)	33 (75.0)	32 (91.4)	
**BMI**	30.2 (22.7–55.7)	31.0 (18.7–52.2)	28.2 (21.5–51.7)	0.40
**HTN**	16 (66.7)	30 (68.2)	28 (80.0)	0.41
**DM**	7 (29.2)	7 (15.9)	8 (22.9)	0.43
eGFR Preop *	55.8 (20.6–112.6)	46.6 (17.4–99.0)	57.1 (18.89–106.0)	0.22
**Tumor Size (cm)**	2.9 (1.4–5.8)	4.3 (1.6–12.5)	3.2 (1.5–6.3)	**<0.001**
**RENAL Score**	6.5 (4–10)	8.5 (5–11)	7 (4–10)	**0.002**
**Surgical Approach**				
Robotic	11 (45.8)	0 (0)	24 (68.6)	**<0.001**
Open	13 (54.2)	44 (100)	11 (31.4)	
Operative Time *	206 (66–419)	210 (90–335)	209 (67–339)	0.99
**Renorrhaphy**				
**1 Layer**	8 (36.4)	9 (23.1)	12 (35.3)	0.65
**2 Layers**	13 (59.1)	29 (74.4)	21 (61.8)	
**Other/None**	1 (4.6)	1 (2.6)	1 (2.9)	
**T-Stage**				
T1	16 (66.7)	17 (38.6)	28 (80.0)	**<0.001**
T2	6 (25.0)	15 (34.1)	7 (20.0)	
T3	2 (8.3)	12 (27.3)	0 (0)	
**Histology**				
ccRCC	18 (75.0)	33 (75.0)	23 (65.7)	0.33
pRCC	3 (12.5)	1 (2.3)	6 (17.1)	
chRCC	0 (0)	2 (4.6)	1 (2.9)	
Other	3 (12.5)	8 (18.2)	5 (14.3)	
**Parenchyma Pathology**				
Healthy	15 (65.2)	17 (43.6)	18 (58.1)	0.22
Unhealthy	8 (34.8)	22 (56.4)	13 (41.9)	
Weeks to Postop Scan *	7 (2–70)	5 (2–67)	7.5 (2–21)	0.16
**Resection Volume**				
Parenchyma Resected	26.1 (0–717.3)	16.77 (0–1730.7)	86.3 (0–1373.4)	**<0.001**
Parenchyma Change *	−40.0 (−486.6, 129.5)	−76.2 (−625.0, 121.8)	−32.4 (−204.2, 181.6)	0.21
**Postop Dialysis**				
No	23 (95.8)	36 (81.8)	33 (94.3)	0.15
Yes	1 (4.2)	8 (18.2)	2 (5.7)	

Abbreviations: body mass index (BMI); hypertension (HTN); diabetes mellitus (DM); estimated glomerular filtration rate (eGFR); renal nephrometry score (RENAL); clear cell, papillary, and chromophobe renal cell carcinoma (ccRCC, pRCC, and chRCC). * indicates median (IQR). Values are frequencies (percentages) for categorical variables and medians (ranges) for continuous variables, with *p*-values from Chi-Square tests (Fisher’s Exact where appropriate) and Wilcoxon tests. **Bold** represents significance with *p*-value < 0.05.

## Data Availability

The data presented in this study are available on request from the corresponding author.
